# Functional Food Potential of White Tea from East Black Sea Region: Targeting GREM1 Expression and Metabolic Dysregulation in Obesity

**DOI:** 10.3390/ijms27020929

**Published:** 2026-01-16

**Authors:** Mehtap Atak, Hülya Kılıç, Bayram Şen, Medeni Arpa

**Affiliations:** 1 Department of Medical Biochemistry, Faculty of Medicine, Recep Tayyip Erdoğan University, Rize 53100, Türkiye; mehtap.atak@erdogan.edu.tr (M.A.); hulya.kilic@erdogan.edu.tr (H.K.); 2 Laboratory of Biochemistry, Education and Research Hospital, Recep Tayyip Erdoğan University, Rize 53100, Türkiye; byrmsn06@gmail.com

**Keywords:** Gremlin1, GREM1, BMP4, obesity, insulin resistance, white tea

## Abstract

Obesity is a major global health concern, being associated with insulin resistance and multiple metabolic disorders. Gremlin 1 (GREM1), a bone morphogenetic protein (BMP) antagonist, is increasingly recognized as a key regulator of adipose tissue dysfunction and impaired thermogenesis in obesity. Orlistat, a lipase inhibitor that reduces dietary fat absorption, is one of the most commonly used pharmacological agents for obesity management. White tea has demonstrated antioxidant and anti-obesity properties in experimental models. The aim of this study was to evaluate the effects of white tea on metabolic parameters (HOMA-IR, BMP4, Gremlin1) and GREM1 expression in rats made obese by a high-fat diet (HFD). A total of 40 male Sprague-Dawley rats were randomized into five groups: a standard diet group (STD); a high-fat diet group (HFD); an HFD + orlistat group (ORL); an HFD + 50 mg/kg white tea group (WT50); and an HFD + 150 mg/kg white tea group (WT150). Obesity was induced by feeding the rats a 45% high-fat diet for 3 weeks. Serum insulin, glucose and HOMA-IR levels were measured. Levels of GREM1 and BMP4 in serum and retroperitoneal adipose tissue were assessed. White tea supplementation significantly reduced weight gain and HOMA-IR compared to the HFD group. GREM1 mRNA expression in visceral adipose tissue decreased markedly in the WT50 and WT150 groups (*p* = 0.002 and *p* = 0.017, respectively). Serum GREM1 levels were significantly lower in the white tea-treated groups than in the HFD group (*p* = 0.011). Tissue BMP4 levels were only significantly reduced in the WT50 group (*p* = 0.005), indicating a non-linear dose–response pattern. There was a negative correlation between serum BMP4 levels and weight gain (rho = −0.440, *p* = 0.015). White tea was associated with improvements in metabolic parameters in an HFD-induced obesity model. These observations suggest a potential association between white tea bioactives and adipose tissue-related molecular pathways implicated in obesity. Given the short intervention duration and the exploratory design of this animal study, the findings should be interpreted with caution.

## 1. Introduction

In recent decades, the global prevalence of obesity has increased dramatically, primarily due to increasingly sedentary lifestyles, urbanization, and the overconsumption of high-calorie foods. Excessive accumulation of adipose tissue resulting from a chronic imbalance between energy intake and expenditure plays a central role in the development of metabolic syndrome and related disorders [1]. The economic burden of healthcare expenditure related to obesity continues to escalate worldwide, highlighting the urgent need for effective preventive and therapeutic strategies [2].

Adipose tissue is recognized as a highly dynamic and metabolically active organ. It is recognized two types of adipose tissue [3]. The first type is white adipose tissue (WAT), which secretes a variety of adipokines, cytokines and hormones that influence appetite, glucose metabolism and the secretion of inflammatory molecules [4]. The second type is brown adipose tissue (BAT), particularly abundant in newborns but has also been identified in adult humans, primarily in the supraclavicular and perirenal regions [2]. Recently, a third population of thermogenic adipocytes has been identified: ‘beige’ or ‘brite’ cells [5,6,7].

Tea is one of the most widely consumed beverages in the world. It is derived from the leaves of the *Camellia sinensis* plant. Depending on the degree of fermentation and processing methods, tea can be broadly classified into several types, including green, black, oolong and white. White tea is considered the least processed form [8]. This processing preserves its high content of bioactive compounds, particularly catechins and their derivatives, such as epigallocatechin gallate (EGCG), epicatechin (EC) and epigallocatechin (EGC) [9]. These compounds are renowned for their potent antioxidant and anti-inflammatory properties [10]. Although white tea is mainly produced in regions with suitable climatic conditions, including China, India, Sri Lanka, and Türkiye.

Studies have demonstrated that tea catechins exhibit a broad spectrum of biological activities, including anti-carcinogenic, cardioprotective, anti-obesity and anti-diabetic effects [11,12,13,14,15]. Studies also have suggested that white tea can reduce lipid accumulation in adipocytes, downregulate pro-inflammatory cytokines, and enhance glucose uptake in insulin-resistant models [16,17,18]. Furthermore, drinking white tea has been associated with improvements in endothelial function, lipid profile and body weight regulation [19]. Given these effects, white tea is considered a promising natural agent in the management and prevention of lifestyle-related disorders, including obesity and type 2 diabetes mellitus [20].

Gremlin 1 (GREM1) is a highly conserved, secreted glycoprotein that functions as an antagonist of bone morphogenetic proteins (BMPs) [21]. It primarily targets BMP2, BMP4 and BMP7. By binding directly to these ligands, GREM1 prevents them from interacting with their receptors [22]. This inhibits the small mothers against decapentaplegic homologs 1, 5, and 8 (SMAD1/5/8) signalling cascade downstream and suppresses the transcriptional activation of peroxisome proliferator-activated receptor gamma (PPARγ), a key regulator of adipocyte differentiation [23,24]. GREM1 thus plays a critical role in preserving the phenotype of white adipocytes while limiting the thermogenic browning process associated with improved metabolic flexibility and energy expenditure [25]. BMPs can also activate non-SMAD pathways, such as mitogen-activated protein kinase (MAPK) signaling. Among BMPs, BMP4 plays a key role in the differentiation of mesenchymal stem cells into brown adipocytes and the induction of uncoupling protein 1 (UCP1), a hallmark of mitochondrial thermogenesis [26]. Elevated GREM1 levels in obesity have been shown to impair this process, contributing to reduced energy dissipation and enhanced lipid accumulation [27]. In animal models fed a high-fat diet (HFD), GREM1 expression increases significantly in visceral adipose tissue, which is associated with impaired glucose tolerance and increased insulin resistance, even in the absence of significant weight gain [28,29]. Conversely, GREM1 inhibition, whether genetic or pharmacological, has been reported to restore insulin sensitivity, promote thermogenic gene expression, and reduce adipose tissue inflammation [7]. Circulating and tissue levels of GREM1 are elevated in individuals with obesity, type 2 diabetes mellitus (T2DM), and non-alcoholic fatty liver disease (NAFLD). GREM1 is also particularly enriched in human visceral adipose tissue compared to subcutaneous tissue depots [2,28]. This distribution is strongly associated with increased HOMA-IR, fasting insulin and triglyceride levels, and is negatively correlated with adiponectin and insulin-stimulated glucose uptake [29].

We hypothesized that white tea supplementation would be associated with reduced GREM1 expression and improved metabolic parameters in a high-fat diet–induced obesity model. This study aimed to investigate the effects of white tea on obesity and insulin resistance in rats fed a high-fat diet. Specifically, we examined whether white tea could impact the Gremlin 1 (GREM1) and BMP4 signalling pathway, which is involved in regulating fat tissue function and energy balance. Two different doses of white tea were tested and compared with orlistat, a widely used anti-obesity medication [30]. In this study, we preferred to use a whole white tea extract rather than isolated EGCG to capture the synergistic metabolic effects of its complete phytochemical matrix, including catechins, methylxanthines, and other bioactive constituents that are naturally co-consumed in human diets. To the best of our knowledge, this study represents the first experimental investigation exploring the association between white tea supplementation and the GREM1–BMP4 signaling axis in the context of obesity.

## 2. Results

### 2.1. Weight Gain and HOMA-IR Index

**Figure 1:**
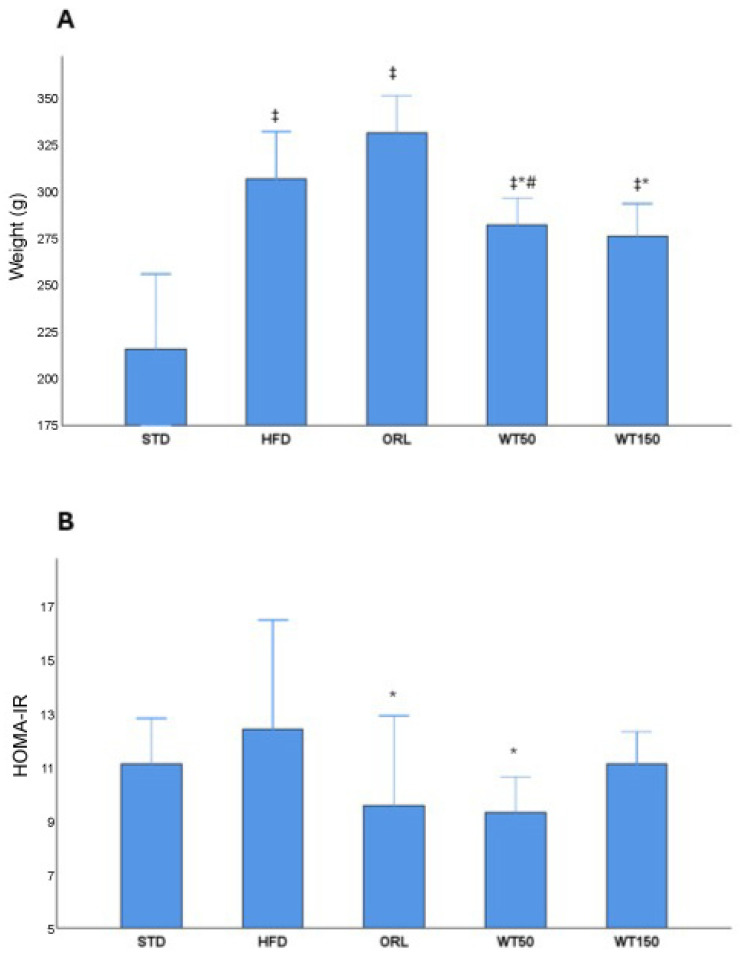
Effects of white tea supplementation on body weight gain and insulin resistance in rats. (**A**) Changes in body weight across experimental groups: standard diet (STD), high-fat diet (HFD), orlistat-treated (ORL), and white tea-supplemented groups (WT50: 50 mg/kg/day; WT150: 150 mg/kg/day). (**B**) HOMA-IR values representing insulin resistance in each group. White tea supplementation reduced HOMA-IR compared to the HFD group, indicating improved insulin sensitivity. *: Significant vs. HFD; #: Significant vs. ORL; ‡: Significant vs. STD.

A metabolic evaluation revealed significant differences between the groups in terms of both the weight gain and HOMA-IR index (Figure 1, Table 1). Rats on a high-fat diet (HFD) demonstrated a significant increase in weight gain compared to the standard diet group (*p* = 0.001, η^2^ = 0.56). The WT50 and WT150 groups gained significantly less weight than the HFD group, with the lowest weight gain observed in the WT150 group (*p* = 0.001, η^2^ = 0.46). The ORL group showed a wide range in weight gain (median 330 g, [290–349 g]), comparable to the HFD group (*p* = 1.000), indicating no clear reduction in weight gain relative to HFD (Figure 1A).

A similar pattern was observed for insulin resistance, as estimated by the HOMA-IR index. The HFD group exhibited the highest HOMA-IR values (median 12.5 [10.6–16.6]). Orlistat administration was associated with a significant but partial reduction in HOMA-IR compared with HFD (median 9.6 [6.2–13], *p* = 0.007; η^2^ = 0.32), although values remained elevated compared with STD. Furthermore, the WT50 group demonstrated reduced HOMA-IR values compared with HFD (median 9.35 [9.1–10.7], *p* = 0.011; η^2^ = 0.32), indicating an improvement in insulin sensitivity. The WT150 group showed only a modest reduction vs. HFD (median 11.2 [9.9–12.4], *p* = 1.000) that did not reach statistical significance (Figure 1B) (Table 1).

### 2.2. GREM1 Expression in Visceral Adipose Tissue

**Figure 2:**
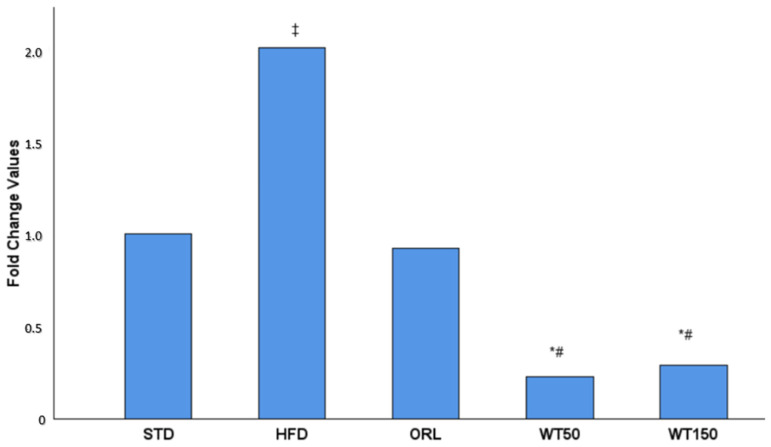
Effect of white tea supplementation on GREM1 gene expression in adipose tissue. Fold change values of *GREM1* mRNA expression in the standard diet (STD), high-fat diet (HFD), orlistat-treated (ORL), and white tea-supplemented groups (WT50: 50 mg/kg/day; WT150: 150 mg/kg/day) are shown. GREM1 expression was markedly increased in the HFD group compared to the STD group, while both white tea-treated groups exhibited a pronounced reduction in GREM1 levels, comparable to orlistat treatment. Data are presented as mean fold change relative to the STD group. *: Significant vs. HFD; #: Significant vs. ORL; ‡: Significant vs. STD.

In the quantitative real-time PCR analysis, the expression of GREM1 in visceral adipose tissue was evaluated as a fold change relative to the non-obese control group. As illustrated in Figure 2, GREM1 expression was approximately two-fold higher in the high-fat diet (HFD) group than in the control group. In contrast, expression levels in the WT50 and WT150 groups were significantly lower than in the HFD group (*p* = 0.002 and *p* = 0.017, respectively). Furthermore, the WT50 group exhibited significantly lower expression than the ORL group (*p* = 0.037).

GREM1 expression was evaluated by qRT-PCR and is presented as fold change relative to the STD group, which was normalized to 1.00. A 2.00-fold increase was observed in the HFD group. The ORL group showed a value close to baseline (0.92-fold). In contrast, the white tea-treated groups demonstrated marked reductions, with 0.23-fold in the WT50 group and 0.29-fold in the WT150 group.

### 2.3. Serum and Tissue Protein Levels of GREM1 and BMP-4

**Figure 3:**
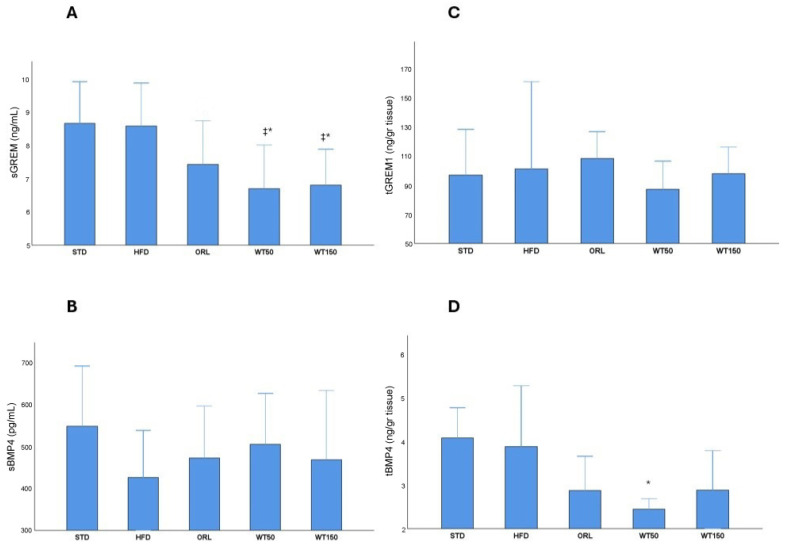
Serum and tissue levels of GREM1 and BMP4 across experimental groups. Box plots show sGREM1 (**A**), sBMP4 (**B**), tGREM1 (**C**) and tBMP4 (**D**) concentrations in rats from the STD, HFD, ORL, and white tea-treated groups. *: Significant vs. HFD; ‡: Significant vs. STD.

Analysis of serum and tissue biomarkers revealed notable group-specific variations. Serum Gremlin-1 (sGREM1) concentrations were significantly higher in the STD and HFD groups (median: 8.7 ng/mL, 8.1–9.9, and median: 8.6 ng/mL, 7.0–9.9, respectively) than in the treatment groups. Notably, the WT50 and WT150 groups exhibited markedly lower sGREM1 levels than the HFD and STD groups, with statistical significance (*p* = 0.011 and *p* = 0.010, respectively; η^2^ = 0.29). The ORL also showed lower sGREM1 levels (7.4 ng/mL (6.8–8.8)), though this reduction was not statistically significant when compared with the HFD group (Figure 3A). There was no significant difference in serum BMP-4 (sBMP4) levels between groups (*p* = 0.611), with median values ranging from 426 pg/mL (269–537) in the HFD group to 504 pg/mL (366–625) in the WT50 group. This suggests that circulating BMP-4 concentrations were not substantially affected by the interventions (Figure 3B). Tissue levels of Gremlin-1 (tGREM1) and BMP-4 (tBMP4) exhibited distinct profiles. Although tGREM1 concentrations were higher in the HFD (101.5 ng/g, 83.9–161.7) and ORL (108.4 ng/g, 60.3–127.0) groups than in the STD (97 ng/g, 81.5–128.7), WT50 (87.3 ng/g, 85.4–106.8) and WT150 (98.0 ng/g, 50.9–116.5) groups, these differences were not statistically significant (*p* = 0.383) (Figure 3C). Tissue BMP-4 concentrations were higher in the STD and HFD groups (median: 4.9 ng/g, 2–4.8, and median: 3.9 ng/g, 3.1–5.3, respectively). Notably, tissue BMP-4 levels were significantly lower only in the WT50 group (2.45 ng/g, 2.03–2.69) than in the HFD group (3.9 ng/g, 3.12–5.3), with *p* = 0.005 (η^2^ = 0.34), indicating a tissue-specific regulatory effect of low-dose white tea. This effect was not observed in the WT150 group, whereas WT150 produced a greater reduction in body weight gain but a less pronounced improvement in HOMA-IR. These findings suggest a complex, non-linear dose–response relationship that requires further investigation. No other significant differences in tBMP4 were observed between the remaining groups (Figure 3D).

### 2.4. Correlation Analysis Among Weight Gain, GREM1, and BMP4

Correlation analysis revealed a significant negative association between serum BMP-4 levels and weight gain (rho = −0.440, *p* = 0.015). A moderate positive correlation was observed between sGREM1 and tGREM1 levels (rho = 0.387, *p* = 0.035). Additionally, sGREM1 levels were positively correlated with tBMP4 levels (rho = 0.560, *p* = 0.001) (Table 2).

Overall, the metabolic and molecular findings show a coherent but outcome-specific pattern. Reductions in body weight gain and improvements in insulin resistance were accompanied by alterations in circulating and tissue-level markers related to the GREM1–BMP4 axis; however, these associations were not uniformly dose-dependent. While changes in serum protein levels measured by ELISA were partially mirrored by tissue-level measurements, gene expression data obtained by qPCR did not always parallel protein-level alterations. The negative correlation observed between serum BMP4 levels and weight gain, as well as the positive correlation between serum GREM1 and tissue BMP4 levels, were not anticipated a priori. These associations are reported as observational findings and may reflect complex regulatory interactions between systemic metabolic status and local adipose tissue signaling rather than direct causal relationships. These observations highlight a complex relationship between systemic metabolic outcomes and local molecular regulation in adipose tissue.

## 3. Discussion

The experimental data from our study suggests a potential involvement of the role of GREM1 as a central regulator in the pathophysiology of obesity, as reported in the literature. The data also show that the metabolic improvement effects of white tea administration may be explained by changes in GREM1 levels. These findings suggest that white tea may contribute to the modulation of adipose tissue-specific molecular responses, with GREM1 potentially acting as a mediator or target structure in this process. Increased GREM1 gene expression in visceral adipose tissue, alongside the highest serum GREM1 levels observed in the HFD group, may suggest that GREM1 is activated both locally and systemically in obesity-related pathophysiological processes. This corroborates the previously reported role of GREM1 in adipose tissue dysfunction and metabolic disorders [31]. The significant increase in insulin resistance in the same group may suggest that this increase in GREM1 levels may be associated with metabolic disorders.

White tea supplementation was associated with changes in components of this molecular axis, with more pronounced effects observed at the lower dose. Significant decreases in GREM1 gene expression and serum levels were observed in the white tea-treated group, alongside a notable reduction in weight gain and insulin resistance. The reduction in weight gain and enhanced insulin sensitivity observed with white tea supplementation in our study align with prior research indicating that green tea preparations promote weight loss and support weight management [32,33,34]. These results imply that white tea could restrict adipocyte hypertrophy and enhance systemic insulin sensitivity by inhibiting GREM1 production. It has indeed been demonstrated in the literature that white tea catechins, particularly EGCG, modulate Wnt and BMP signalling pathways, reducing oxidative stress and inflammation [35,36]. In this context, it can be suggested that the metabolic improvement observed in our study was due to white tea suppressing GREM1-BMP4 antagonism. Given the multifaceted effects of GREM1 on regulating adipogenesis, angiogenesis, and immune response processes, the effects of white tea on this protein may not be limited to lipid metabolism but also may extend to the structural and functional components of the adipose tissue microenvironment. In particular, the antagonistic effect of GREM1 on BMP4 may inhibit the maturation of adipocyte progenitors and the development of healthy adipose tissue, which may in turn pathologise the expansion of adipose tissue and trigger adverse processes such as hypertrophy, hypoxia and inflammation [27]. Depletion of GREM1 by white tea may have broken this chain and promoted more balanced adipocyte differentiation and tissue remodelling. However, GREM1 is produced not only by adipocytes, but also by fibroblasts, endothelial cells, and immune cells in the stromal vascular fraction of adipose tissue [37,38]. Consequently, the white tea supplementation may have exerted a far-reaching regulatory effect on all cellular components of adipose tissue, including not only fat cells. The effects of GREM1 on these cellular subunits are associated with alterations in tissue-level fibrosis, extracellular matrix (ECM) remodelling, and local immune responses. Suppressing this axis contributes to maintaining adipose tissue function.

Administration of white tea at a dose of 50 mg/kg was associated with improvements in parameters related to this pathological process. White tea administration was associated with lower GREM1 expression and serum levels, accompanied by reductions in weight gain and insulin resistance. Thus, white tea may provide not only symptomatic improvement, but also play a direct regulatory role in the GREM1-BMP4 axis by targeting obesity-related molecular dysregulation. Previous studies have reported that white tea polyphenols, particularly the EGCG compound, have regulatory effects on the Wnt/BMP signalling pathways and reduce oxidative stress and inflammation. To the best of our knowledge, our study is the first to demonstrate that these effects are exerted through GREM1 in a concrete model, making it possible to evaluate white tea not only for its antioxidant properties, but also as an active biological agent that can modulate gene and protein expression in adipose tissue. EGCG, one of the major catechins found in white tea, has been described as a potent biological agent capable of modulating multiple signalling pathways at the cellular level [39]. The differential effects observed between WT50 and WT150 across molecular and metabolic endpoints suggest that the dose–response relationship of white tea is not linear. The reasons underlying this divergence remain unclear and warrant further investigation in studies specifically designed to address dose dependency.

In human clinical and epidemiological studies, regular consumption of green or white tea rich in EGCG has been associated with reductions in body weight, visceral fat, and serum triglyceride levels, as well as improved insulin sensitivity and antioxidant capacity [40,41,42]. While the anti-adipogenic, anti-inflammatory and antioxidant effects of EGCG in obesity models are well documented, our study provides new evidence that these effects may be mediated through the GREM1-BMP4 axis [43]. While BMP4 acts as a critical morphogen for physiological adipogenesis, GREM1 is a protein that directly antagonizes this pathway. Therefore, EGCG’s effect on enhancing BMP4 signalling or promoting GREM1 inhibition may play a decisive role in adipogenesis and adipose tissue plasticity. In our study, the significant decrease in BMP4 levels observed in the WT50 group following white tea administration suggests that this effect may be related not only to GREM1 suppression, but also to a rebalancing effect at the systemic level. Furthermore, EGCG has been reported to affect histone acetylation and DNA methylation at an epigenetic level, thereby permanently altering the expression profiles of adipogenesis-related genes [44]. These findings suggest that white tea may support long-term as well as temporary metabolic remodelling.

Previous comparative studies have reported that white tea exhibits equal or even stronger antioxidant and metabolic regulatory activities than other tea types, such as green, oolong, or black tea. This superior bioactivity has been attributed to its minimal processing, which preserves high levels of unoxidized catechins, particularly EGCG. Therefore, the favorable outcomes observed in our study may be partly related to the higher catechin content and antioxidant potential of white tea compared to more oxidized tea varieties. Although the present study primarily focused on the association between white tea administration and GREM1 expression, it is plausible that additional mechanisms contribute to the observed metabolic improvements. White tea is known to contain abundant catechins, especially EGCG, which have been reported to influence lipid and glucose metabolism, enhance antioxidant capacity, and improve mitochondrial function. These effects may collectively support the protective metabolic response observed in our model. Future studies incorporating a broader molecular analysis are warranted to further elucidate these pathways [45,46,47].

Orlistat was included as a pharmacological comparator due to its established efficacy in reducing fat absorption and improving metabolic parameters. Consistent with its known mechanism of action, orlistat administration improved insulin resistance but did not significantly alter GREM1 expression or circulating levels, suggesting that its metabolic effects are largely independent of the GREM1–BMP4 axis. In contrast, white tea supplementation was associated with alterations in GREM1- and BMP4-related parameters, although direct mechanistic differences cannot be inferred from the present data. These observations suggest that white tea and orlistat may influence metabolic outcomes through distinct and potentially complementary pathways, a hypothesis that warrants further investigation.

## 4. Materials and Methods

### 4.1. Experimental Animals and Study Groups

In the study, male rats were preferred to avoid the confounding hormonal variations in the estrous cycle that may affect metabolic and gene expression parameters. 40 male Sprague-Dawley rats weighing 150–200 g and 6–8 weeks aged were first subjected to a 1-week acclimation period. After the adaptation period, the rats were randomly divided into five groups. Randomization was performed using a simple randomization method. All investigators involved in treatment administration, outcome assessment, and laboratory analyses (qPCR and ELISA) were blinded to group allocation, and samples were analyzed using coded identifiers. The STD group acted as the control group and was fed only a chow diet. The HFD group was fed a high-fat diet. The ORL group was fed a high-fat diet and given orlistat once they had become obese. The WT50 group was fed a high-fat diet and given 50 mg/kg/day of white tea once they had become obese. The WT150 group received a high-fat diet and 150 mg/kg/day of white tea after becoming obese. All groups except the STD group were fed a high-fat diet ad libitum until obesity was established, defined as a ≥20% increase in body weight. Following obesity induction, which lasted a total of 16 weeks, the respective interventions (white tea or orlistat) were administered while dietary conditions were maintained. The composition of the high-fat diet (Arden Research & Experimental Co., Ankara, Türkiye) provided 45% of total energy from fat. The STD group received a chow diet (Bayramoğlu Feed, Erzurum, Türkiye) (Table 3). In this study, orlistat was included solely as a pharmacological reference control to contextualize weight-related outcomes, given that its mechanism, gastrointestinal lipase inhibition, differs fundamentally from the intracellular signaling pathways targeted by white tea. After obesity was induced, the rats were given a chow diet for three weeks. Animals were housed in standard polycarbonate cages (50 × 70 cm) under controlled environmental conditions (22 ± 2 °C, 55 ± 10% humidity, 12 h light/dark cycle), with six rats per cage. Standard laboratory bedding was used, and animals had ad libitum access to food and water throughout the study. Food intake was monitored at the cage level. Body temperature was measured with a rectal probe and maintained at 36–37 °C during the procedure. Food and water were provided ad libitum. The rats’ weights were measured weekly, and a weight gain of at least 20% was accepted as a criterion for obesity [48]. During this period, they were administered 30 mg/kg orlistat and two different doses of white tea (50 mg/kg and 150 mg/kg) via oral gavage. At the end of the experiment, the rats were sacrificed after 12 h of fasting. Anesthesia was induced by intraperitoneal injection of 50 mg/kg ketamine hydrochloride (Ketalar, Pfizer Ltd., Istanbul, Türkiye) and 10 mg/kg xylazine hydrochloride (Rompun, Bayer, Whippany, NJ, USA). Blood samples were collected at the time of sacrifice and processed immediately. Serum was separated by centrifugation, and samples were visually inspected to exclude hemolysis prior to analysis. Tissue samples were excised, rinsed in cold phosphate-buffered saline, and homogenized immediately after collection using the specified homogenization buffer. Homogenization and subsequent biochemical analyses were performed without prolonged storage or freeze–thaw cycles. Therefore, no protease inhibitor cocktail was added, as immediate processing minimized the risk of proteolytic degradation. All analyses were conducted promptly following sample preparation. All animals were treated according to the protocols for the care and use of laboratory animals under the guidelines of the National and International Research Council. The phenolic composition of white tea was analyzed by Hüner Yiğit M. et al. using HPLC-DAD methodology, demonstrating a diverse profile of bioactive catechins and polyphenols [19]. Among the catechins, epigallocatechin (EGC) and epigallocatechin gallate (EGCG) were identified as the most abundant compounds in the white tea extract. The white tea used in this study was obtained from ÇAYKUR (General Directorate of Tea Enterprises, Rize, Türkiye), produced from the first spring flush harvested in May. As white tea can be collected only once per year due to its strict leaf-bud selection criteria, ÇAYKUR manufactures a single annual batch, which undergoes minimal processing according to traditional white tea production practices. The harvested buds are only withered and dried, without rolling or oxidation steps, and subsequently packaged in glass jars for consumer use. The product used in this study belonged to the 2020 production year; however, individual batch numbers are not assigned by the manufacturer. For experimental procedures, the dried white tea was extracted by steeping at 80 °C for 10 min in distilled water at a ratio of 1 g per 20 mL, followed by sequential filtration through sterile gauze and paper filters. The filtrate was standardized to dosing concentrations (50 mg/kg and 150 mg/kg) and administered via oral gavage. Orlistat was administered orally by gavage at a dose of 30 mg/kg once daily. Orlistat was freshly prepared in distilled water immediately prior to administration and was obtained from commercially available capsules (Xenical^®^). The solution was administered once daily by oral gavage using a stainless steel, ball-tipped gavage needle designed for rodents (16-gauge). The selected dose and administration protocol were based on previously published experimental studies demonstrating the metabolic efficacy of orlistat in rodent models of diet-induced obesity [30]. The catechin and polyphenol composition of this white tea, including the predominance of EGC and EGCG, has been previously profiled using HPLC-DAD methodology [19]. Given the unique agricultural and processing characteristics of ÇAYKUR white tea—harvested only once annually and minimally processed—our findings apply specifically to the 2020 spring-flush white tea used in this study.

### 4.2. Preparation of White Tea Samples

White tea leaves were obtained from a single commercial supplier (General Directorate of Tea Enterprises (ÇAYKUR) in Rize, Türkiye) and originated from the same production batch to ensure consistency in composition. The leaves were minimally processed and dried at low temperature to preserve catechin content. The leaves were harvested during the first flush only, in May, in line with ÇAYKUR’s standard practice. White tea is not harvested during later flushes. The preparation and dose selection of white tea were based on previous studies [30]. Before administration, the samples were cooled to room temperature and delivered to the subjects by oral gavage. Experimental groups received 1 mL/day of the substances at the same time daily. Subjects in the other groups received 1 mL/day of water by gavage.

### 4.3. Preparation of Blood and Tissue Specimens

Serum samples were collected by centrifugation of blood collected from the subjects for 15 min at 1500× *g* at 2–8 °C after waiting for clotting. For homogenization of the obtained retroperitoneal adipose tissue samples, 1 mL homogenization buffer was prepared with 20 mM sodium phosphate + 140 mM potassium chloride at pH 7.4. 1 mL of homogenization buffer was added to 0.1 g of tissue and homogenized [31]. After homogenization, centrifugation was performed at 800× *g* for 10 min at 2–8 °C.

### 4.4. Analysis of Samples

Gremlin 1 (Cat: SG-21730; SinoGeneClon Biotech Co., Ltd., Hangzhou, China), BMP 4 (Cat: SG-20681; SinoGeneClon Biotech Co., Ltd., Hangzhou, China) and insulin (Cat: SG-20161; SinoGeneClon Biotech Co., Ltd., Hangzhou, China) levels were analyzed by the ELISA method. All assays employed rat-specific antibodies pre-coated onto 96-well microplates. Standards and appropriately diluted samples were incubated with HRP-conjugated detection antibodies, followed by tetramethylbenzidine (TMB) substrate addition. The enzymatic reaction was terminated with the stop solution, and absorbance was measured at 450 nm using a microplate reader. Analyte concentrations were calculated from standard curves generated by serial dilutions of the provided standards. The detection ranges were 0.75–15 mIU/L for insulin, 0.2–9 ng/mL for Gremlin-1, and 8–400 pg/mL for BMP-4, with sensitivities of 0.2 mIU/L, 0.05 ng/mL, and 2.2 pg/mL, respectively. Intra-assay and inter-assay coefficients of variation were <8% and <10%, respectively, in accordance with the manufacturer’s specifications. The HOMA-IR index was calculated using the formula glucose x insulin/405. Total RNA was isolated using a kit (High Pure RNA Isolation Kit, Roche, Mannheim, Germany), and its concentration was determined using µdrop plates on Thermo Multiskan Go (Thermo Fisher Scientific, Waltham, MA, USA). The High-Capacity cDNA Synthesis Kit was used to generate cDNA (Applied Biosystems, San Francisco, CA, USA) from total RNA (1000 ng RNA/20 µL reaction). We performed qRT-PCR in a 96-well optical plate using LightCycler 480 Probes Master in a Roche Lightcycler 480 II (Roche, Mannheim, Germany), diluting the master mix with nuclease-free water. A total of 30 ng of cDNA was used in each qRT-PCR reaction (20 µL) containing 1 µL. The following probes were purchased from Thermo: Grem1 (Cat: 4448489) and GAPDH (Cat: 4448489). GAPDH gene expression was used as a reference for normalizing the results.

### 4.5. Statistical Analyses

Statistical analyses were performed using IBM SPSS Statistics, v23.0 (SPSS Inc., Chicago, IL, USA) and Microsoft Office Excel. GREM1 expression was evaluated by fold change. Within-group distributions were reported as frequencies (n, %). Given the small sample size and the absence of normal distribution assumptions, non-parametric statistical tests were selected for group comparisons. The Kruskal–Wallis test with post hoc Dunn’s test with Bonferroni correction was used to assess differences between groups for continuous numerical variables. Effect size estimates were calculated for Kruskal–Wallis analyses using η^2^ to quantify the magnitude of group differences. Confidence intervals were not systematically reported, as the statistical analyses were based on non-parametric methods within an exploratory preclinical framework, where inference focused on hypothesis testing rather than interval estimation. Spearman correlation analysis was performed to evaluate the relationship between the data, and significant rho values were determined. Data are presented as median and minimum–maximum (min-max). *p* < 0.05 was considered significant.

## 5. Conclusions

In summary, the primary finding of this study was the association between white tea supplementation and alterations in GREM1 expression in a high-fat diet–induced obesity model. Secondary outcomes included changes in BMP4-related parameters, insulin resistance as assessed by HOMA-IR, and body weight gain. Notably, these effects did not follow a consistent linear dose–response pattern, with divergent responses observed between the WT50 and WT150 groups across different endpoints. Importantly, the effects of white tea were outcome- and dose-dependent but not linear: WT50 was associated with a significant reduction in BMP4-related parameters, whereas WT150 was not, while WT150 resulted in a greater reduction in body weight gain but a less pronounced improvement in HOMA-IR. Overall, the findings provide exploratory evidence that white tea supplementation is associated with improvements in selected metabolic parameters and related molecular markers in this experimental setting. However, direct regulatory effects or therapeutic applicability cannot be inferred from this short-term animal study. Moreover, as the phytochemical composition of white tea may vary according to geographic origin, cultivar, harvest season, and processing conditions, the present results should be interpreted as specific to the ÇAYKUR spring-flush white tea used herein. Although direct measurements of total fat mass and detailed adipose tissue morphology were not performed, obesity is a multifactorial condition characterized by both metabolic and molecular alterations. In this context, the observations reported here may serve as a basis for future hypothesis-driven studies. Further long-term and mechanistic investigations incorporating comprehensive metabolic phenotyping are required to clarify the role of white tea–derived compounds in obesity-related pathways.

Several limitations of this study should be acknowledged. First, the use of an animal model limits the direct generalizability of the findings to humans. Second, the intervention duration was relatively short (3 weeks), which may limit the assessment of long-term metabolic and molecular effects. Food intake, energy expenditure, adipose tissue histology, and thermogenic markers (such as UCP1 and PGC-1α) were not assessed, precluding a more comprehensive evaluation of energy balance mechanisms and tissue-level correlates of the molecular findings. The sample size was determined within the framework of an exploratory animal study and was not based on an a priori power calculation, and the observed effects did not follow a consistent linear dose–response pattern between WT50 and WT150. Furthermore, while gene expression and circulating levels of GREM1 and BMP4 were evaluated, direct confirmation of their protein expression within adipose tissue was not performed. It should also be noted that the phytochemical composition of white tea may vary substantially depending on geographic origin, cultivar, harvest season, and processing conditions; therefore, the findings of the present study should be interpreted in the context of the East Black Sea region–derived white tea used herein and may not be directly generalizable to all white tea preparations. Finally, the exclusive use of male rats precluded the assessment of sex-specific effects, and additional metabolic and inflammatory biomarkers (such as HbA1c, adipokines, and inflammatory mediators) were not included. Collectively, these limitations highlight the need for future studies incorporating longer intervention periods, comprehensive phytochemical characterization, mechanistic analyses, and broader metabolic phenotyping.

## Figures and Tables

**Table 1:** Results of the analysis of biochemical parameters among the treated groups.

	HFD	ORL	WT50	WT150	*p* Value *
	Median (Min–Max)	Median (Min–Max)	Median (Min–Max)	Median (Min–Max)	
sGREM (ng/mL)	8.6 (7–9.9)	7.4 (6.8–8.8)	6.7 (6.4–8)	6.8 (5.6–7.9)	0.011
sBMP4 (pg/mL)	426 (269–537)	472 (347–595)	504 (366–625)	468 (361–632)	0.611
tGREM1 (ng/gr tissue)	101.5 (83.9–161.7)	108.4 (60.3–127)	87.3 (85.4–106.8)	98 (50.9–116.5)	0.383
tBMP4 (ng/gr tissue)	3.9 (3.12–5.3)	2.875 (2.37–3.67)	2.45 (2.03–2.69)	2.89 (1.98–3.8)	0.005
HOMA-IR	12.5 (10.6–16.6)	9.6 (6.2–13)	9.35 (9.1–10.7)	11.2 (9.9–12.4)	0.007
Weight gain (gr)	305 (297–330)	330 (290–349)	281 (252–295)	275 (250–292)	0.001

*: Kruskal–Wallis test.

**Table 2:** Correlations between GREM1/BMP4 levels, HOMA-IR, and weight gain in rats.

		tGREM1	tBMP4	sGREM	sBMP4	HOMA-IR
tBMP4	rho	0.315				
sGREM	rho	0.387 *	0.560 **			
sBMP4	rho	0.022	0.145	0.212		
HOMA-IR	rho	−0.118	0.143	0.229	0.112	
WeightGain	rho	0.158	−0.008	−0.049	−0.440 *	0.101

*: <0.05, **: <0.001.

**Table 3:** The compositions of the diet.

Company	Name/Code	Content	
		Ingredient	Amount/Calories
Bayramoğlu Yem ve Un Sanayi Tic. A.Ş. (Erzurum, Türkiye)	Control feed	Humidity	12.8%
		Raw protein	23%
		Raw fat	1.7%
		Raw cellulose	3.7%
		Raw ash	8.3%
		Sodium	0.5%
		Vitamin A	12,000,000 IU/kg
		Manganese (manganese sulfate)	95 mg/kg
		Iron (iron sulfate monohydrate)	31 mg/kg
		Zinc (zinc oxide)	95 mg/kg
		Cobalt (cobalt carbonate)	0.5 mg/kg
		Selenium (sodium selenite)	0.3 mg/kg
		Iodine (calcium iodate anhydrous)	2.28 mg(kg
Arden Araştırma Deney (Ankara, Türkiye)	High-fat diet feed (22% fat, 20% protein, total 4500 kcal)	Casein	200 g/kg (800 kcal)
		Corn starch	109 g/kg (410.9 kcal)
		Dextrinized starch	193 g/kg (780 kcal)
		Sugar	121.5 g/kg (486 kcal)
		Palm oil	220 g/kg (1980 kcal)
		Cellulose	50 g/kg
		Mineral mixture (S10026)	10 g/kg
		Vitamin mixture (V10001)	10 g/kg (40 kcal)
		L-cystine	3 g/kg
		Choline bitartrate	2.5 g/kg (12 kcal)
		DCP (dicalcium phosphate)	13 g/kg
		Calcium carbonate	5.5 g/kg
		Potassium citrate monohydrate	16.5 g/kg
		Methyl paraben	0.014 g/kg
		Aromatic chemicals	48 g/kg

## Data Availability

All data supporting the findings of this study are included in the article.
